# A New Colorimetrically-Calibrated Automated Video-Imaging Protocol for Day-Night Fish Counting at the OBSEA Coastal Cabled Observatory

**DOI:** 10.3390/s131114740

**Published:** 2013-10-30

**Authors:** Joaquín del Río, Jacopo Aguzzi, Corrado Costa, Paolo Menesatti, Valerio Sbragaglia, Marc Nogueras, Francesc Sarda, Antoni Manuèl

**Affiliations:** 1 SARTI Research Group, Electronics Department, Universitat Politècnica de Catalunya (UPC), Rambla de la Exposición 24, Vilanova i la Geltrú-Barcelona 08800, Spain; E-Mails: Marc.nogueras@upc.edu (M.N.); antoni.manuel@upc.edu (A.M.); 2 Instituto de Ciencias del Mar (ICM-CSIC), Paseo Maritimo de la Barceloneta, 37-49, Barcelona 08003, Spain; E-Mails: sbragaglia@icm.csic.es (V.S.); siscu@icm.csic.es (F.S.); 3 Consiglio per la Ricerca e la Sperimentazione in Agricoltura, Via della Pascolare, Monterotondo Scalo 16-00015, Rome, Italy; E-Mails: corrado.costa@entecra.it (C.C.); paolo.menesatti@entecra.it (P.M.)

**Keywords:** coastal fishes, cables observatories, OBSEA, automated video-imaging, colorimetric calibration, swimming rhythms, 3D Thin-Plate Spline warping

## Abstract

Field measurements of the swimming activity rhythms of fishes are scant due to the difficulty of counting individuals at a high frequency over a long period of time. Cabled observatory video monitoring allows such a sampling at a high frequency over unlimited periods of time. Unfortunately, automation for the extraction of biological information (*i.e.*, animals' visual counts per unit of time) is still a major bottleneck. In this study, we describe a new automated video-imaging protocol for the 24-h continuous counting of fishes in colorimetrically calibrated time-lapse photographic outputs, taken by a shallow water (20 m depth) cabled video-platform, the OBSEA. The spectral reflectance value for each patch was measured between 400 to 700 nm and then converted into standard RGB, used as a reference for all subsequent calibrations. All the images were acquired within a standardized Region Of Interest (ROI), represented by a 2 × 2 m methacrylate panel, endowed with a 9-colour calibration chart, and calibrated using the recently implemented “3D Thin-Plate Spline” warping approach in order to numerically define color by its coordinates in n-dimensional space. That operation was repeated on a subset of images, 500 images as a training set, manually selected since acquired under optimum visibility conditions. All images plus those for the training set were ordered together through Principal Component Analysis allowing the selection of 614 images (67.6%) out of 908 as a total corresponding to 18 days (at 30 min frequency). The Roberts operator (used in image processing and computer vision for edge detection) was used to highlights regions of high spatial colour gradient corresponding to fishes' bodies. Time series in manual and visual counts were compared together for efficiency evaluation. Periodogram and waveform analysis outputs provided very similar results, although quantified parameters in relation to the strength of respective rhythms were different. Results indicate that automation efficiency is limited by optimum visibility conditions. Data sets from manual counting present the larger day-night fluctuations in comparison to those derived from automation. This comparison indicates that the automation protocol subestimate fish numbers but it is anyway suitable for the study of community activity rhythms.

## 1. Introduction

Field measurements of the swimming activity rhythms of rocky fishes are scant due to the difficulty of counting individuals at a high frequency over a large period of time [1]. Poor access to repeated sampling at statistically relevant intervals and frequencies limits temporal studies of fauna, impeding establishment of a solid linkage between perceived biodiversity and species behavior [2]. Such kinds of studies are of relevance for the development of models predicting fish community changes in spite of changing environmental conditions, involving human and climatic stressors [3].

Technological improvements in coastal fish monitoring would require the development of a new observational technology capable of acquiring data sets at a high frequency over long temporal durations (from week to years) [4]. This technology is now available, being represented by cabled video-observatories [5]. Cabled seafloor observatories are multiparametric platforms connected to the shore for power and real-time data transmission that often carry video cameras in addition to sensors measuring habitat conditions [6]. These allow the researcher to monitor biotic activities at different levels of complexity (from the individual animal, to population, species up to the level of the whole community), often providing real-time online access allowing the observer to view current events [7,8].

Unfortunately, major drawbacks in using still cabled observatories cameras chiefly refer to the need for manual processing of very large sets of images for animal detection, counting and when required, classification [9]. That drawback can only be overcome by implementing suitable automated-video imaging protocols, which have recently been customized for the study of activity rhythms with video-cabled observatories of aphotic deep-sea areas [9–11]. Such an effort has not yet been attempted in the shallow coastal zones, where the greater variability in environmental illumination and often complex background substrates (e.g., reefs or coarse bottoms) consistently complicate the elaboration of efficient protocols [2].

The customization of an automated protocol for the 24-h video-counting at a frequency of minutes has not yet been implemented in shallow water coastal cabled observatories, being of potential relevance also for other coastal platforms worldwide, such as for example the Martha's Vineyard Coastal Observatory [12] of Massachusetts' Katama Air Park and the Long-term Environmental Observatory [13] in New Jersey. Accordingly, automated video-imaging protocols for fish detection, coping with the difficulties of working at depth zones where light levels vary markedly in relation to the day-night cycle are of relevance, since development in more challenging scenarios in comparison to the more disphotic deep-sea [2]. In this study, we describe the customization and functioning of a new automated video-imaging protocol for the day-night continuous counting of fishes (with no classifications) within a standardized field of view. Our protocol was developed to work with time-lapse photographic outputs proceeding form still coastal cabled observatory cameras. Our objective was to test its monitoring capabilities under markedly different environmental illumination conditions, in order to promote a discussion on feasibilities and limitations of automated video-imaging in coastal areas, as a reliable tool to monitor fish swimming rhythms at different temporal scales.

## 2. Experimental Section

### 2.1. The Platform and the Panel for the Field of View Standardization

**Figure 1.**
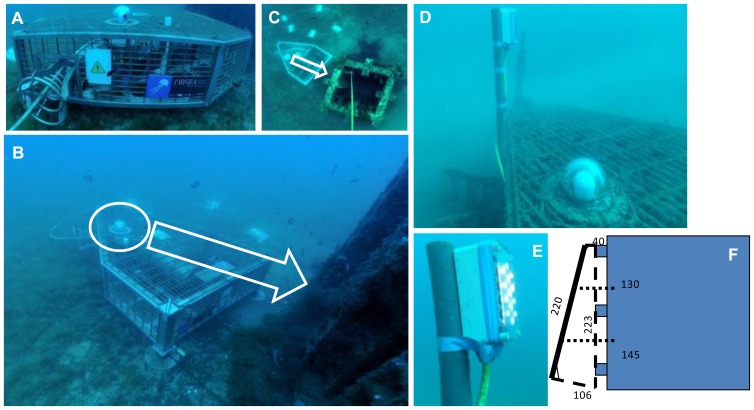
Different details of the OBSEA cabled video-observatory, the lighting system and the artificial reef with the appended ROI panel. (**A**) lateral view of the OBSEA platform with a detail of the cable and the external structure; (**B**) up-side view, where the circle indicates the position of the video camera (the arrow represents the direction in which images were acquired in relation to the artificial panel deployed aside the reef); (**C**) a top view of the OBSEA, in which are visible the artificial reef and the observatory together (see **B** for arrow meaning); (**D** and **E**), indicate respectively the positioning of the LED light arrays in relation to the camera (only one is visible, being the other on the back of the photographer) and a particular of its structure when ON; and finally, (**F**) Scheme depicting the installation of the panel (that will be the ROI acquired by the camera) aside the artificial reef (number are distances in cm).

The expandable SEAfloor OBservatory (OBSEA; www.obsea.es) is a multiparametric cabled video-platform located at 20 m depth 4 km off Vilanova i la Geltrú (Catalonia, Spain) in front of an artificial reef [14,15]. It is endowed with an OPT-06 Underwater IP Camera (OpticCam; Ocean Presence Technologies, Santa Cruz, CA, USA, Figure 1A,B), which can acquire digital images of the environment surrounding the OBSEA at 360° with a resolution of 640 × 480 pixels (Mpeg/Mjpeg; 18 × optical zoom). An artificial barrier is located at 3 m distance from the camera (Figure 1C). In the recent past, the OBSEA camera has be efficiently used for manual monitoring of the fish community at a high frequency and over prolonged periods of time, but only with daytime images [16].

The OBSEA was recently implemented with a nocturnal lighting system (Figure 1D) consisting of two white light LED arrays (Figure 1E), in order to allow fish counting over the 24-h cycle in a continuous fashion. Each array consisted of 13 high-luminosity white LEDs with a total power of 30 W and generated an emission power of 3,800 lumens (49 μmole/m^2^/s) along the maximum light propagation vector at an angle of 38°. The two lights illuminated a panel at the constant Region of Interest (ROI) for fish counting (Figure 1F), installed aside the artificial reef, from one meter rear the camera.

**Figure 2.**
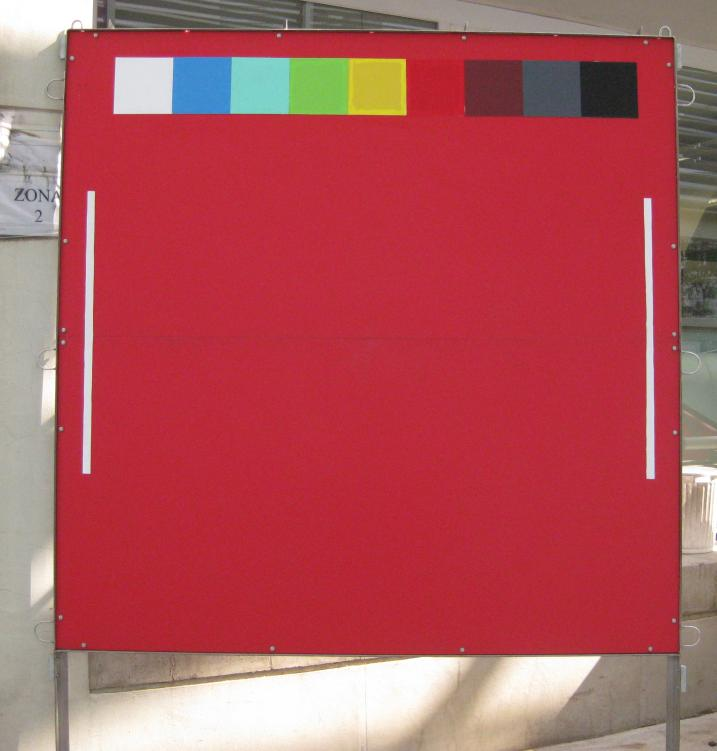
The methacrylate red panel used for automated RGB colorimetric calibration of images taken by OBSEA camera at different times of the day. White vertical lines (100 cm length) can be used as general size bars for fish length determination in absence of more precise measuring methods (*i.e.*, lasers).

The camera always aimed at 45° angle toward a red methacrylate homogenously panel of 220 × 220 cm, approximately 2 m above the seabed (Figure 2), installed next to the artificial reef. Its presence and uniform colouring were required to standardize automated video-imaging within a constant ROI up to a maximum extent, given the variable lighting conditions, as usually occurring in coastal areas. In particular, the panel provided a constant framework for fish counting, when considering that the average visibility at OBSEA can be very variable according to local turbidity [16], considering turbidity as the cloudiness of water caused by individual particles (suspended solids).

A 9-colour chromatic chart was also installed on the panel upper side (see Figure 2), in order to allow image calibration for Red, Green and Blue (RGB) contents at different time of the day and under different environmental illuminations (see Section 2.3). RGB calibration was required for the image thresholding, which is a critical initial step for fish presence identification [9].

### 2.2. The Time-Lapse Photographic Acquisition

Images were acquired over 30-min periods during 18 days (22 October to 9 November 2011 starting and ending at 0:00 h and 21:30 h local time, respectively). A procedure controlling the ON-OFF status of the lighting immediately before and after image acquisition at night was implemented, since constant lighting at video-monitoring may disrupt behavioural observations [9,17], due to fish avoidance or attraction [18,19]. The lights activation was automatically controlled by a customized LabVIEW application that also manages the camera white balance. The automated protocol for the ON and OFF light switching started and ended 2 s prior and after respectively, the camera image acquisition.

### 2.3. The Automated Video-Imaging Protocol for Fish Counting

All automation procedures were implemented in a Matlab 7.0 environment (Image Processing Toolbox). The spectral reflectance values for each of the 9-colour chart patches of the panel (see Figure 2) was measured in the visible range (between 400 and 700 nm wavelengths with a step of 10 nm) prior immersion, using a Portable Integrated-Sphere D50/2 Spectrocolorimeter (Xrite, SP64, Grandville, MI, USA). The obtained reflectance of each patch was then converted in standard RGB (sRGB) value, using the Matlab OptProp Freeware Toolbox., according to [20]. These converted values were used as a reference for all calibration treatments of underwater images at filtering (as required for fish identification by thresholding).

**Figure 3.**
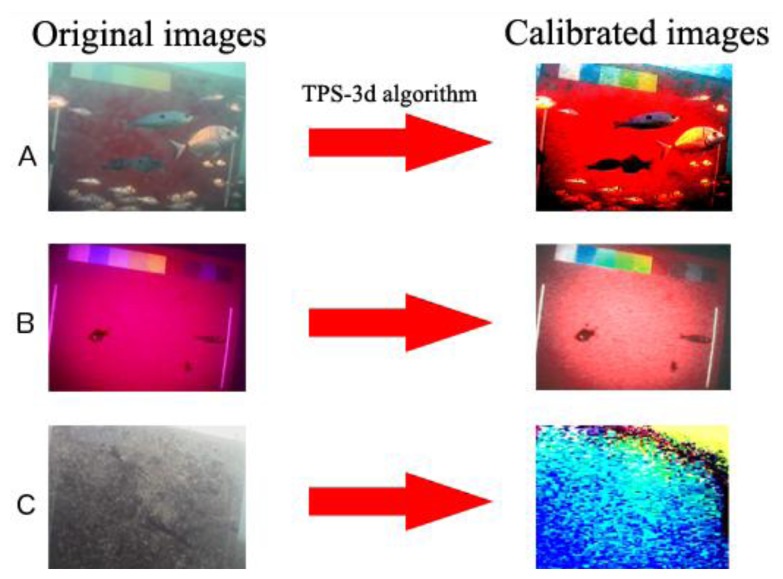
Example of three images with different illumination conditions (**A**, day, **B**, night; **C**, day with turbidity), before and after the calibration.

All the images were calibrated using the recently implemented “3D Thin-Plate Spline” warping approach [21] (Figure 3). For each calibrated image, the mean RGB values of a 100 × 100 pixels area in the centre of each patch were extracted. That operation was repeated on a subset of 614 images as a training set, manually selected since of optimum visibility conditions. RGB values of a ROI encompassing the central portion of the panel from all images (so including the 614 used as training set) were ordered altogether through Principal Component Analysis (PCA) in order to verify if calibration could be automatically used to classify good from bad images. That represents a necessary preliminary step in automated video-imaging to avoid the wasting computational time and potential results noise.

Fish automated counting was then carried out only on the 614 images a ROI encompassing the central portion of the panel was selected. On all the RGB channels a Euclidean distance was calculated from each pixel with respect of the background (*i.e.*, the mean value for the 100 × 100 pixels panel central area). Basing on these distances a segmentation algorithm based on the Roberts edge detection has been applied. The Roberts operator performs a simple 2-D spatial gradient measurement on an image [22]. It thus highlights regions of high spatial gradient, which often correspond to edges. In the output, pixel values at each point represent the estimated absolute magnitude of the spatial gradient of the input image at that point.

### 2.4. Data Treatment of Video-Imaging Fish Counts

The time series of automated and manual total fish counts were firstly represented in the domain of time. Both data sets were then treated by the same time series analysis tools, in order to generally evaluate the efficiency of automation in a statistic fashion. Firstly, both series were screened by Chi-Square periodogram analysis [23] between 660 and 1,500 min (equals to 11-h and 25-h, respectively) [1]. Periodogram analysis was run with El Temps software [24]. In the periodogram output plots, the highest peak crossing the significance (*p* < 0.05) threshold represented the maximum percentage of total data variance explained by the inherent dominant periodicity. Periodicity was indicated by that peak value. We also reported the % of variance for each detected significant periodicity, being that measure proportional to the rhythm strength in time series outputs [25,26].

At this point, we carried out a compared waveform analysis on the automated (*i.e.*, merged data) and manual visual detections data sets. A mean fluctuation over a standard period of 24-h (*i.e.*, the waveform) was calculated in order to identify time zones where automation increases its rate of failure. In order to do so, each data set was partitioned into subsets of 24-h duration. An average diel fluctuation was then computed by averaging fish counts values of all sub-sets at corresponding timings. The phase was then computed according to the Midline Estimating Statistic of Rhythm (MESOR) method [27]. The MESOR value was estimated by re-averaging all waveform values and representing the result as a threshold line on the waveform plot. All mean values above the line defined a significant increment in visual counts. The onset and offset of activity were estimated by considering the first and the last value above MESOR, respectively. Also, the percentage of activity in both waveforms was compared at daytime as marker of signal goodness.

We also considered the intensity variations (scale from 0 to 255) of the Green channel (G) of the green colour chart patch (see Figure 2), in order to compare automated video-imaging performance with local conditions of illumination and turbidity. The waveform analysis was again carried out on the G dataset, in order to assess the timing where most difficulties in automation occurred for the presence of turbidity (drops in G intensity). To the resulting waveform plot, we superimposed the total counting of discharged images by 30 min, as an ulterior parameter of evaluation.

## 3. Results

**Figure 4.**
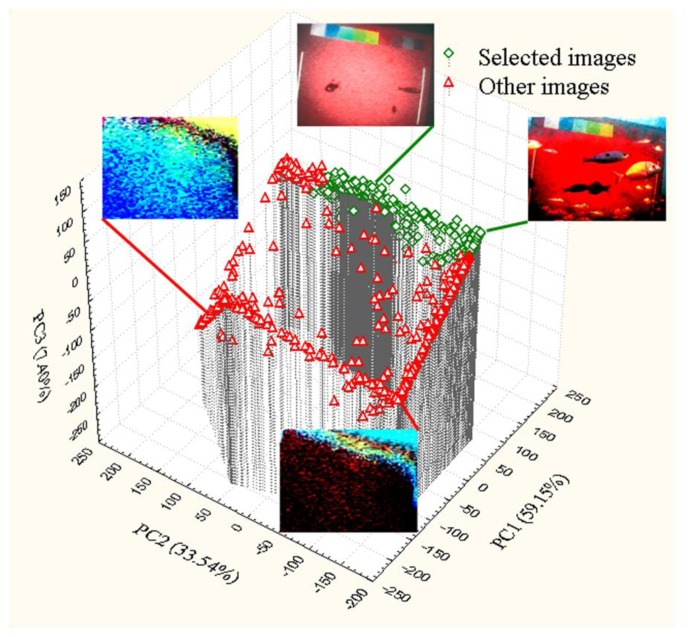
PCA outputs on the mean calibrated RGB values of a 100 × 100 pixels area in the centre of the panel. In green the selected images; in red the discarded images. Around the graph are represented four examples of calibrated images (selected and discarded according to their relative positioning in the PCA output (clockwise: night-time good, daytime good; night bad, day bad).

In this study we acquired continuously 908 images corresponding to 18 days at 30 min frequency. A total number of 614 images (67.6%) were selected for the further analyses according to the “3D Thin-Plate Spline” the calibration procedure. The PCA ordination of the mean calibrated RGB values of a 100 × 100 pixels area in the center of the panel is reported in Figure 4. It is possible to observe that the selected images (in green) are positioned altogether on the positive side of PC1 and PC3. That calibration method efficiently allowed the selection of the images to be further processed discarding all the others (*i.e.*, those presenting bad illumination conditions as well as too elevated turbidity).

**Figure 5.**
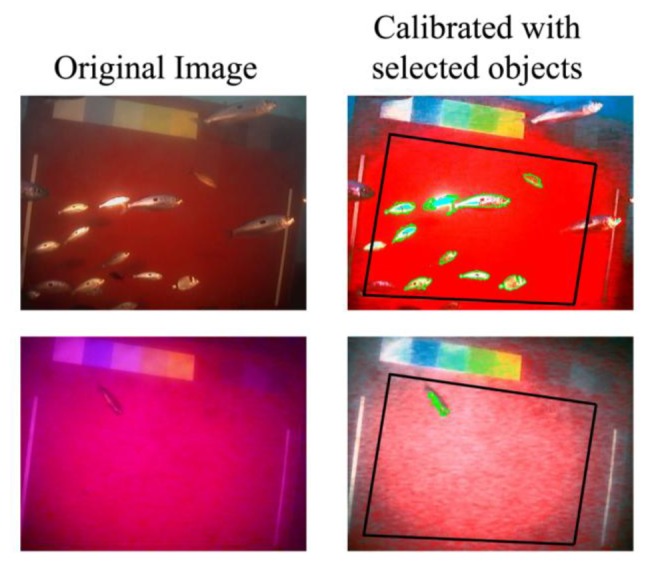
Examples automated processing of time-lapse images acquired by the OBSEA coastal cabled observatory at both day and night-time. Two examples of original images (on the left; above day, below night) and their chromatically calibrated outputs (on the right) were reported as an example of fish identification performance. The black polygon represents the ROI and selected object selected by the Roberts edge algorithm within it are evidenced with a green outline.

In Figure 5 four examples of images processed with the automated protocol were reported. It is possible to observe how the two images in the A block were processed with good performances of object extraction, meanwhile the B block reported images processed with fair performances (object overlapping on the upper side and object not recognized on the bottom side).

**Figure 6.**
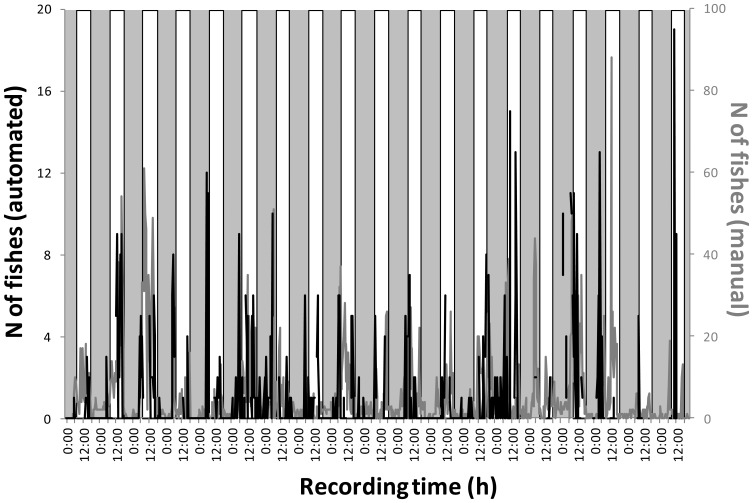
Time series outputs for automated (black) and manual (grey) time series in fish video counts as obtained continuously at day and night-time, with 30 min time-lapse photographic frequency sampling carried out during one month at the OBSEA cabled observatory.

The comparison between automated and manual fish count sets (Figure 6) can be used to generally evaluate the goodness of the method. Automated time series show a similar phasing in fish count increases at daytime than manual ones, although for different levels. In fact, total detected fishes are equals to 678 for the automated protocol *vs.* 4,751 for the manual counting. That difference was given by the minor number of images considered suitable for automation processing according to the criteria established in Figure 4.

**Figure 7.**
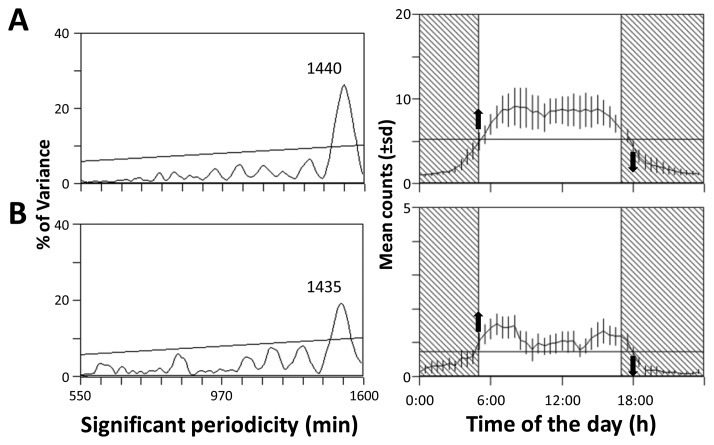
Periodograms (left) and waveforms (right) for visual (**A**) and automated (**B**) fish counts time series as reported at OBSEA. In waveforms plots the dashed vertical rectangle depicts the average night duration during the whole video sampling period. MESOR is the horizontal bar in waveforms (A = 5.21; B = 0.74) along with ONSET (upper arrow; the first values above MESOR) and OFFSET (lower arrow; the first value below MESOR) timings.

Despite abundance differences produced using the automated *vs.* the manual fish counting similar diel periodicities could be detected. This can be considered as an indication of the viability of the implemented protocol for automated fish counting, from the point of view of activity rhythms estimations by cabled observatories. Periodogram screening of both automated and manually-generated data sets (Figure 7A) indicated significant and very similar periodicities (approx. 1,440 min equals 24 h). According to differences in reported abundances by the two methods, the rhythmicity in time series show also a differential strength as indicated by the peak amplitude (*i.e.*, the % of variance): automated, 19.1%; manual, 26.2%.

At the same time, waveform analysis (Figure 7B) revealed a marked similar diurnal phases with a consistent nocturnal drop for both data sets (*i.e.*, equals time of ONSET and OFFSET of count increases in relation to the MESOR). Also, the area percentage at daytime was very similar indicating a similar community activity profile: automated, 73.4%; manual, 76.0%. Anyway, one should be notice how phases amplitude are different (given overall differences in abundance).

**Figure 8.**
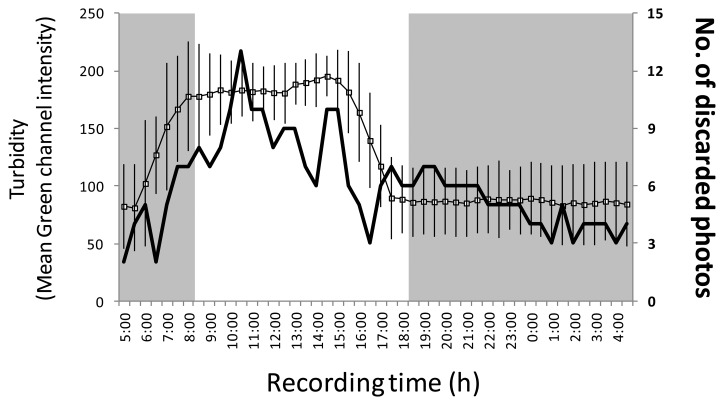
The influence of turbidity on the elaborated automated video-imaging protocol over a standard 24-h cycle, as indicated by turbidity as quantified through the averaged Green content (green-channel) and total discharged photos. Grey area is the night.

The automated processing showed to be influenced by turbidity, which was more apparent in daytime images (Figure 8). The Green content of images was used as a proxy of suspended particles matter. That increased during the day for the effect of downward incoming solar light enhancing the reflection of that matter. At night times such an effect was consistently diminished for the action of the two lights, strongly illuminating the ROI in the horizontal plane. The number of images discarded during daytime hours was higher than the ones at night times. Accordingly, overall detections were more different during the day, as timing coinciding with the larger augments of fishes in the OBSEA area.

## 4. Discussion

In this study, we customized a new automated video-imaging protocol to count fishes at day and night in a continuous fashion over a standardized ROI. We implemented a colorimetric calibration procedure that could efficiently discriminate suitable images for fish counting, as shown by PCA ordination (see Figure 4). That preliminary screening was required for the too variable light and turbidity conditions usually experienced in the OBSEA coastal areas [5,16]. Once calibration was carried out, the Roberts edge algorithm could discriminate fish present with the ROI. Globally, results indicate that automation efficiency is limited by optimum visibility conditions, being procedures for image quality evaluation prior the recognition of moving fishes also a key step to be carefully taken into account.

Periodogram and waveform analysis outputs for automated and manual data sets are similar (see Figure 7), although quantified parameters in relation to the strength of respective rhythms were different. Clearly, data sets derived from manual counting presented the larger day-night fluctuations being reported rhythmic stronger in comparison to those derived from automation. This comparison indicates that our automation protocol subestimate fish numbers but it is anyway suitable for the study of community activity rhythms.

Automation in the processing of cabled observatory video materials must be customized according to each location, being substrate, depth and hence overall photic conditions greatly variable [2]. Here, we tried to standardize our automated video processing to a maximum extent, by adding a constant and colorimetrically uniform ROI. In that manner, fish counts are at least homogenized in relation to the depth of the field of view. Under these conditions, activity patterns were anyway resolvable in a fashion similar to outputs provided by manual counting. This indicates that activity rhythms in the community can be studied by automated video-imaging because image discarding and the derived counting underestimation are constantly occurring through consecutive days. Swimming rhythms were measurable at a community level, since image discarding occurred more frequently at daytime, when fish counts were anyway at their maximum in the study areas [16].

Image acquisition occurred in a time-lapse mode at a 30 min frequency, with a light ON duration of few seconds. As already pointed out in video imaging studies with artificial lighting ON at night-time or constantly ON during the 24-h as in the deep-sea [11,19,28], attraction or repulsion in fishes may occurs after few seconds. In particular, Doya *et al.* [29] estimated a suitable time of light attraction within the first 25 s of light ON at video recording in the deep-sea. Presently, an estimation of behavioural alterations on the local fish community produced by our photographic sampling schedule at nighttime is not available. Anyway, activity rhythms were detected and fish counts were very low at nighttime as usually confirmed also by other sampling methods (e.g., visual census; [1]). Accordingly, we here confirm that a 30 min time lapse mode in photographic acquisition with associated and intermittent lighting ON at night time is not perturbing the recording of community rhythms and hence the overall study of fish behavior.

## 5. Conclusions/Outlook

Marked variations in coastal fish counts were detected with daily periodicity by an automated video-monitoring carried out with the OBSEA cabled observatory. This fact alone justifies the effort of developing increasingly more efficient methods for the remote, autonomous, and long-lasting monitoring of marine animals communities. That socio-economical and scientific need is now summarized by the fast developing “cabled observatory science” [7]. In this monitoring, automation in video imaging plays a key role, since cameras are the only sensor allowing the extraction of biological information at the complex ecological scale of animals and their communities [2,9]. Suitable automation may contribute to transform cabled observatories into permanent ecosystem monitoring tools [6–8,11], fulfilling the goals of major ongoing infrastructural projects of relevance for the future of European marine research [30–32].
